# Pelagic *Sargassum* community change over a 40-year period: temporal and spatial variability

**DOI:** 10.1007/s00227-014-2539-y

**Published:** 2014-09-14

**Authors:** C. L. Huffard, S. von Thun, A. D. Sherman, K. Sealey, K. L. Smith

**Affiliations:** 1 Monterey Bay Aquarium Research Institute, 7700 Sandholdt Rd, Moss Landing, CA 95039 USA; 2 Coastal Ecology Laboratory, Biology Department, University of Miami, Coral Gables, FL 33124 USA

## Abstract

Pelagic forms of the brown algae (Phaeophyceae) *Sargassum* spp. and their conspicuous rafts are defining characteristics of the Sargasso Sea in the western North Atlantic. Given rising temperatures and acidity in the surface ocean, we hypothesized that macrofauna associated with *Sargassum* in the Sargasso Sea have changed with respect to species composition, diversity, evenness, and sessile epibiota coverage since studies were conducted 40 years ago. *Sargassum* communities were sampled along a transect through the Sargasso Sea in 2011 and 2012 and compared to samples collected in the Sargasso Sea, Gulf Stream, and south of the subtropical convergence zone from 1966 to 1975. Mobile macrofauna communities exhibited changes in community structure and declines in diversity and evenness within a 6-month time period (August 2011–February 2012). Equivalent declines in diversity and evenness were recorded in the same region (Sargasso Sea, 25°–29°N) in 1972–1973. Recent community structures were unlike any documented historically, whether compared to sites of the same latitude range within the Sargasso Sea, or the broader historical dataset of sites ranging across the Sargasso Sea, Gulf Stream, and south of the subtropical convergence zone. Recent samples also recorded low coverage by sessile epibionts, both calcifying forms and hydroids. The diversity and species composition of macrofauna communities associated with *Sargassum* might be inherently unstable. While several biological and oceanographic factors might have contributed to these observations, including a decline in pH, increase in summer temperatures, and changes in the abundance and distribution of *Sargassum* seaweed in the area, it is not currently possible to attribute direct causal links.

**Electronic supplementary material:**

The online version of this article (doi:10.1007/s00227-014-2539-y) contains supplementary material, which is available to authorized users.

## Introduction

Pelagic forms of the brown algae (Phaeophyceae) *Sargassum* are a defining characteristic of the Sargasso Sea. Ranging from approximately 20°–40°N and 30°–75°W (Butler et al. 1983; Coston-Clements et al. 1991; Laffoley et al. 2011), the Sargasso Sea is bordered by the Gulf Stream to the west, north equatorial current to the south, and weak circulation to the east (Siegel et al. 2001). Although this floating ecosystem is located in the center of the oligotrophic North Atlantic Gyre (Coston-Clements et al. 1991), it has higher concentrations of metazooplankton (Riemann et al. 2011) and phosphorous (Culliney 1970) than the surrounding waters. The Sargasso Sea is bisected by the subtropical convergence zone (STCZ), a frontal region which is often associated with increased productivity (Andersen et al. 2011). *Sargassum* floating on the surface ranges in size from fist-sized clumps to massive rafts tens of meters in diameter (Marmorino et al. 2011) forming contiguous features possibly extending 100 miles or more (Carr 1986).

Two floating species of *Sargassum*, *S. natans* and *S. fluitans*, have dominated algal collections in the Sargasso Sea (Parr 1939; Fine 1970; Niermann 1986). Both species have buoyant, gas-filled bladders and are considered pelagic in origin, reproducing by vegetative fragmentation (Butler et al. 1983). *Sargassum* macroalgal rafts provide important feeding and spawning grounds for pelagic fishes (Casazza and Ross 2008) and seabirds (Trott et al. 2010) and are a high-productivity ecosystem along open-ocean (oligotrophic) migratory paths of endangered humpback whales and sea turtles (Trott et al. 2010). *Sargassum* rafts have been designated as “essential fish habitat,” a status that impacts international management recommendations for the Sargasso Sea (South Atlantic Fishery Management Council 2002).

The first comprehensive studies of *Sargassum* abundance in the Sargasso Sea were conducted between 1933 and 1935 (Parr 1939). More than 40 years later, another quantitative study of *Sargassum* was conducted in overlapping areas between 1977 and 1981 (Stoner and Greening 1984). Stoner reported a major decrease in *Sargassum* compared to the 1930s study and attributed this decline to either natural or anthropogenic causes. Subsequent recalculations found that there was no significant decline in *Sargassum* biomass except in two sampling events in the southwestern Sargasso Sea (Butler and Stoner 1984). Recently, satellite imagery has revealed that the distribution and abundance of *Sargassum* features in the Atlantic are highly variable over space and time (Gower and King 2011; Gower et al. 2013). In 2011, ocean-scale buildup of *Sargassum* biomass was 200-fold higher than the previous 8-year average (Gower and King 2011).

Diverse assemblages of organisms are associated with *Sargassum* rafts, including both attached epibionts and mobile fauna, ranging from microbiota to fishes (Winge 1923; Carpenter 1970; Fine 1970; Ryland 1974; Butler et al. 1983). Coston-Clements et al. (1991) reported over 100 species of fishes and four sea turtle species associated with pelagic *Sargassum* and Trott et al. (2010) reported a total of over 145 invertebrate species. Numerous sponges, fungi, bacteria, diatoms, and protists have also been reported (Thiel and Gutow 2005a). Among these counts, ten fish and invertebrate taxa are currently considered endemic to *Sargassum* (Coston-Clements et al. 1991; Laffoley et al. 2011)*. Sargassum* associates in the Sargasso Sea have been documented to be more abundant than in the Gulf Stream (Stoner and Greening 1984) and possibly more diverse than in some other areas with *Sargassum* rafts, such as the East China Sea (Abé et al. 2013), New Zealand (Kingsford and Choat 1985), coastal Ireland (Norton and Benson 1983), and Iceland (Ingólfsson 1995).

Rising concentrations of atmospheric carbon dioxide are resulting in global warming and increased acidity of the ocean (Feely et al. 2004; Orr et al. 2005; Doney et al. 2009), including in the Sargasso Sea (Bates et al. 2012, 2014). These changing conditions might affect *Sargassum* and associated biota. Ocean acidification and the concomitant decrease in carbonate saturation have been correlated to reduced formation rates of calcareous skeletal structures of some pelagic fauna such as mollusks and foraminifera (Fabry et al. 2008; Bednaršek et al. 2014). Decreasing pH has also been related to decreased reproduction and increased mortality in crustaceans including copepods, euphausiids, and decapods (e.g., Yamada and Ikeda 1999; Watanabe et al. 2006; Metzger et al. 2007). By contrast, elevated concentrations of CO_2_ can stimulate the growth of coastal macroalgae, including Phaeophyceae (Wu et al. 2008). Photosynthetic activity by phytoplankton and brown algae can buffer the boundary layer from low-pH conditions (Wootton et al. 2008; Saderne and Wahl 2012). Taken together, these findings suggest that *Sargassum* seaweed might fare better than its faunal associates as the impacts of climate change progress, influencing the community living within *Sargassum* rafts.

Here, we present a structural study of the *Sargassum* community conducted between Bermuda and the Bahamas in winter and summer months in 2011 and 2012 and compare these observations to historical sample data reported from the same area between 1966 and 1975 (Weis 1968; Fine 1970; Butler et al. 1983). Given rising sea surface temperatures (SST) and acidity in the surface of ocean, including the Sargasso Sea (Bates et al. 2012, 2014), we hypothesized that the diverse biotic community associated with *Sargassum* has changed in macrofauna species composition, diversity, and evenness measures when compared to studies conducted 40 years ago. Globally, the magnitude and direction of biogeographic range shifts correspond with that of temperature change (Pinsky et al. 2013), which is northward in the Sargasso Sea (Friedland et al. 2007). Compared to demersal species, pelagic animals are predicted to experience a greater magnitude in range change in response to surface warming (Cheung et al. 2009). As such, we further predicted that the mobile macrofauna community structure (MMCS) of samples from 2011 and 2012 would be similar to those historic samples collected from more southern sites, signaling a northward shift of rafting community associates.

## Materials and methods

### Terminology

We use three terms to refer to varying sizes of floating *Sargassum* biomass. “Clumps” refer to individual strands or small entangled groups of individual strands that vary from fist to dinner-plate size (9–28 cm diameter). “Rafts” refer to a larger aggregation of many clumps, forming a contiguous habitat (meters in extent). A windrow of floating *Sargassum* formed by Langmuir circulation would be an example of an elongate raft. We choose to use this term over the synonymous terms “patch” and “mat” because of its prevalence in the ecological literature about floating, or “rafting” communities (Thiel and Gutow 2005a, b; Gibson et al. 2006; Macreadie et al. 2011). We use the term “features” when discussing floating masses of *Sargassum* large enough to be detected by the satellite MERIS (Gower and King 2011). “Stations” refer to sampling locations. During each cruise, one sample was recorded from each station and used to assess faunal communities.

### Area of investigation

**Fig. 1:**
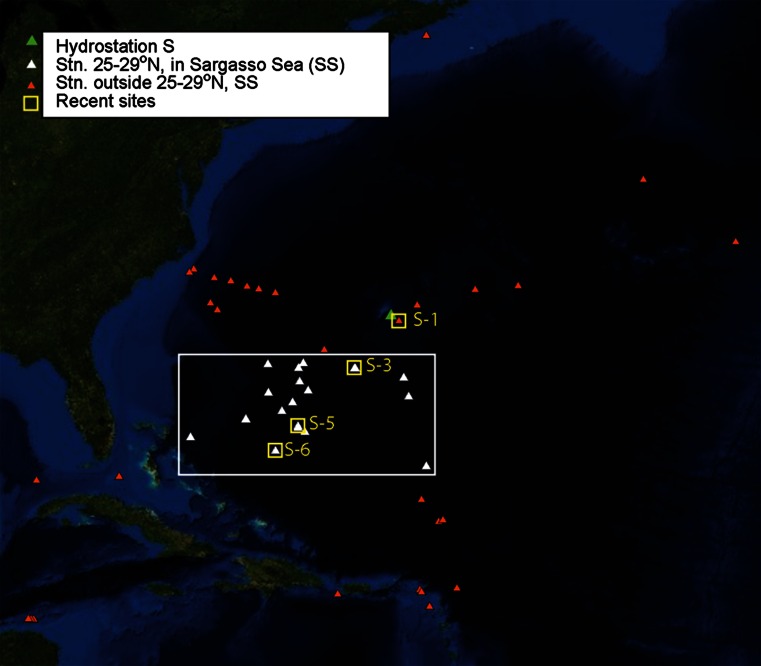
Study area map: stations of recent sample stations (*small yellow boxes*, stations numbered *1*, *3*, *5*, and *6*) and historical sample stations (Weis 1968; Fine 1970; Butler et al. 1983) including Hydrostation S in *green*. Samples used for direct comparison across time series in Sargasso Sea, 25°–29°N (“restricted block”) depicted by white triangles inside larger *white box*. Additional historical stations included in broader analysis of all samples shown as *orange triangles*. Long time-series records of sea surface temperature and pCO_2_ recorded in vicinity of Hydrostation S. Map prepared in ArcMap 10.1

All sampling stations we analyzed are shown in Fig. 1. Our sampling strategy was designed to address fundamental questions concerning variability in Sargassum communities over time and space. We sampled along a transect of stations representative of the central portion of the Sargasso Sea, from Bermuda in the north to the Bahama Islands in the south (Fig. 1; Stations 1, 3, 5, and 6). This transect covered a broad region studied in historical sampling of the *Sargassum* community (Butler et al. 1983; Fine 1970). Our Sta. 1 was located near the long-term time series Hydrostation S (32°N 64°W, shown in Fig. 1 as a green triangle; Bates et al. 2012; Bates et al. 2014). Three cruises on *M/Y Lone Ranger* were conducted over a 13-month period including winter and summer months—February 2011, August 2011, and February 2012—to capture weather extremes that might influence this surface ocean community. Due to weather and failed political clearances, we were only able to sample all four stations during two of the three cruises (February 2011 and February 2012). Mobile macrofauna samples from February 2011 and epibiota samples from August 2011 were compromised after collection and are not reported.

A total of 88 historical sample datasets were analyzed in addition to the recent samples from 2011 and 2012. For brevity, all historical “samples” mentioned throughout refer to the published sample data. We did not have access to the original samples themselves. This total includes 41 samples from Hydrostation S and excludes historical data based on samples with displacement volume (DV) <70 ML (see *Statistical analysis of mobile fauna communities* section below). These stations extended from the continental margin off Nova Scotia to the Windward Islands. Historical sampling dates (ranging from 1966–1975), geographic coordinates of stations, and references for historical sample data are provided in Online Resource 1.

Diversity was found to vary by latitude range in historical samples (see *Statistical analysis of mobile fauna communities* section below). Thus, direct comparisons between recent and historical macrofauna communities were restricted to samples from those stations located within the Sargasso Sea and between 25° and 29°N. We refer to these stations as the “restricted block” (Fig. 1—white triangles in white box). Data based on samples from the restricted block were used to compare MMCS, diversity, evenness, and Multivariate Dispersion Index (MDI) documented in 2011 and 2012 [*N* = 6 (Sta. 3, 5, 6 sampled in 2011 and again in 2012)] to those documented in 1972 (*N* = 2) and 1973 (*N* = 14).

### Oceanographic conditions (SST and pCO_2_)

Sea surface temperature (SST) was recorded during recent cruises by a hull-mounted flow-through Seabird SBE45 thermosalinograph. Recent temperatures represent the SST at the time collections were made. Long-term trends in SST and pCO_2_ at Hydrostation S were available through the Bermuda Atlantic Time series Program (Bates et al. 2012, 2014). These time series data were smoothed to deconvolve seasonality from long-term trends using a negative exponential model (0.7 sampling proportion) in SigmaPlot. Temperature data were available for only 12 other historical stations. We used regression analysis to examine possible correlations between SST and diversity.

### Sampling method

All recent samples were collected from a small boat launched from *M/Y Lone Ranger* to minimize any impact of the large ship on sampling, especially of the mobile species, which can exhibit an escape response. We used standard methods for monitoring mobile macrofauna in the Sargasso Sea (Butler et al. 1983). *Sargassum* clumps were collected in dip nets with a 0.25-m^2^ mouth opening and 505-µm mesh netting. This mesh size is considered sufficient for capturing small macrofauna such as amphipods and gastropods (Tanaka and Leite 1998). Although this method can underestimate fishes (Moser et al. 1998), fishes were included in analyses of diversity, evenness, and community structure because they were nevertheless detected in both recent and historical samples. The dip-netted clumps were placed in 19-L plastic buckets with seawater and then transferred within 30 min to the ship. Each recent sample analyzed here was the combined collection of approximately 10^3^ mL total DV of clumps, collected from a station over the course of 1–2 days. Historical sampling of mobile fauna used this same incremental collection approach, but typically attained lower total DV for each sample. Mobile species in *Sargassum* clump samples were immediately sorted to major taxa at SST. The few specimens of Sargassum fish, *Histrio histrio*, we encountered were quickly photographed and then released back into the surface waters. The residual sample water from each sorting effort, along with a subsequent freshwater rinse, was then filtered through 420-µm screens, and the retained material was preserved for later sorting. Six small aliquots of *Sargassum,* 500–900 mL DV from different individual plants in the full sample, were preserved for later analysis of the attached biota in each sample. The mobile taxa samples, screened material, and *Sargassum* aliquots were preserved in 5 % buffered formalin.

In the laboratory, mobile fauna were sorted, identified to lowest taxon possible, and counted for the samples from August 2011 and February 2012. We compared the diversity and community structure of distinct taxa; however, we could not identify all organisms to the same taxonomic level. Percent coverage by epibiota (attached, sessile growth) on *Sargassum* was assessed in the laboratory using standard photogrammetric techniques (adapted from Bohnsack 1979; see *Epibiota* *percent cover* below) for samples from February 2011 and February 2012.

### Statistical analysis of mobile fauna communities

Using Chao1 analysis (Chao et al. 2009), we verified that sufficient sampling was conducted in 2011 and 2012 to detect >95 % of mobile animal groups predicted to exist at sampling stations at the time of collections. The test statistic *S*_Chao1_ is calculated using the abundance and richness of fauna sampled in relation to the number of taxa represented by only one or two individuals. It can be considered a proxy for understanding sample completeness.

As reported by Fine (1970) for their samples, we found no relationship between sample volume and the number of animal groups detected for the historical station with the largest number of surveys (Hydrostation S: *n* = 41 replicates, DV range 71–1,357 mL, DV vs. number of animal groups detected *r* ^2^ = 0.0918, *F*_1,39_ = 3.9418, *P* > 0.05). This finding suggested that 70 mL was sufficient for capturing the diversity of mobile macrofauna in those historical samples. Based on Chao1 analysis, historical samples with DV <50 mL were estimated to have captured <85 % of animal groups predicted to be present. Thus, we conservatively eliminated from analyses the nine historical samples with a DV of <70 mL, leaving a historical sample size of 88. Displacement volumes for recent samples were much greater than those for historical samples included in analyses, except for Weis (1968), which had a very large sample volume [recent samples: 12.0 ± 2.5 L standard deviation (SD); historical samples except for Weis = 0.3 ± 0.2 L S.D.; Weis 28.3 L].

Analyses of diversity, evenness, MMCS, and MDI were conducted based on treatments of untransformed abundance data in Primer-6 (Clarke and Warwick 2001). Both historical and recent abundance data were standardized to a sample volume of 10^3^ mL, the approximate volume sampled at each station in 2011 and 2012. The DIVERSE routine delivered Shannon diversity (*H*′ log_e_) and Pielou’s evenness (*J*′). Non-metric multi-dimensional scaling (MDS) plots of MMCS were based on Bray-Curtis dissimilarity indices of square-root-transformed abundance data. At least one thousand starts were performed to generate MDS plots, and ANOSIM was used to test for differences in MMCS. RELATE was used to examine cyclicity, and MDI was used to compare degrees of variability between sample groups. The SIMPROF function was performed as part of CLUSTER analyses generated by Primer-6 to examine differences in MMCS of different test groups.

Production and biomass vary considerably along transects in the Sargasso Sea (Riemann et al. 2011), which can complicate statistical testing. Therefore, we divided mobile macrofauna datasets into test groups (latitude range, geographic group, and season) and assessed variability between these groups using historical data. Latitude ranges were 10°–19°N; 20°–24°N; 25°–29°N, and 30°–45°N. One range (25°–29°N) likely encompassed STCZ activity (Riemann et al. 2011), which can have elevated animal abundance and productivity (Andersen et al. 2011). The following geographic groups were used: Gulf Stream, Sargasso Sea, south of the Sargasso Sea, north of the Sargasso Sea, and eastern Sargasso Sea. Seasons were divided as follows: winter (December–February), spring (March–May), summer (June–August), fall (September–November). Preliminary analysis of historical mobile macrofauna abundance and diversity at stations within the Sargasso Sea revealed that the Shannon *H*′ log_e_ diversity index differed by latitude group (Kruskal–Wallis test, *H*_3_ = 16.48, *P* = 0.0009; *H*′ log_e__≤24°N_ = 1.6 ± 0.25; *H*′ log_e__25°–29°N_ = 1.6 ± 0.26; H′ log_e 30°–45°N_ = 1.8 ± 0.25; H′ log_e__30–45°N_ > H′ log_e__10°–24°N_ post hoc Wilcoxon–Mann–Whitney, *U* = 224, *N*_1_ = 14, *N*_2_ = 58, *P* = 0.0048), but not season (Kruskal–Wallis test, *H*_3_ = 4.152, *P* = 0.2455). Therefore, when comparing diversity and evenness of historical and recent samples, we grouped data across all seasons, but separated them by latitude range and geographic area.

Using StatXact-4 and Microsoft Excel, we performed Kruskal–Wallis and analysis of variance (ANOVA) tests to examine differences in mean diversity and evenness across groups, followed by post hoc Wilcoxon–Mann–Whitney *U* tests to examine pairwise differences (Siegel 1956). Kruskal–Wallis and Wilcoxon–Mann–Whitney *U* tests are nonparametric and appropriate for comparisons of groups that have small and/or unequal sample sizes, with non-normal distributions (Siegel, 1956). For pairwise comparisons with small sample sizes (n_1_ < 8), we calculated Mann–Whitney *U* tests by hand using the table of critical values in Siegel (1956).

### Epibiota percent cover

Six strands of *Sargassum* ~50 cm long were chosen haphazardly from each preserved aliquot in February 2011 and February 2012. For each of these subsamples (*n* = 6 per aliquot), we noted the presence or absence of six growth zones described by Ryland (1974). These zones are apical growing tip (zone A), subapical developing blades (zone B), developing bladders (zone C), mature bladders (zone D), mature growth with spots (zone E), and senescent dark brown (zone F). Growth zone E is an advanced growth stage, more prominent in senescent *Sargassum*, while F represents a more senescent growth stage than E. Each strand was placed in a tray and photographed from directly above using a Nikon D200 digital camera mounted on a tripod. Images were cropped so that each photograph represented a single growth zone. Using Image J (Rasband 2012), the macro “Drawrandomdots.txt” (http://rsbweb.nih.gov/ij/macros/DrawRandomDots.txt) was used to overlay at least 250 random dots on each image so that at least 25 dots fell onto the 2-dimensional images of the bladder, stem, and leaf, respectively. We recorded the taxa visible under these dots to estimate percent coverage by epibiota. For epibiont growth forms that projected from the *Sargassum*, such as *Lepas* barnacles or hydroids, we counted the dots that fell where the epibiont attached to the weed, not the projecting portion.

Historical descriptions of epibionts were based on different methods or were qualitative where available for samples in the restricted block, precluding statistical comparisons of sessile *Sargassum* associates across time periods.

### Mobile macrofauna trophic groups

Mobile macrofauna were placed into trophic groups (predators of mobile fauna, predators of sessile fauna, herbivores, and detritivores) according to published food web relationships, feeding observations, and gut content analyses (designations and references in Online Resource 2). Where data were not available for a particular taxon, the trophic group of congeners or the next highest taxonomic level was used. Where trophic group reports were inconsistent, the highest level reported was used. Only those trophic groups representing at least 1 % of all samples were included. As such, salps (which filter feed on plankton) and parasites were excluded.

## Results

### Oceanographic conditions (SST and pCO_2_)

**Fig. 2:**
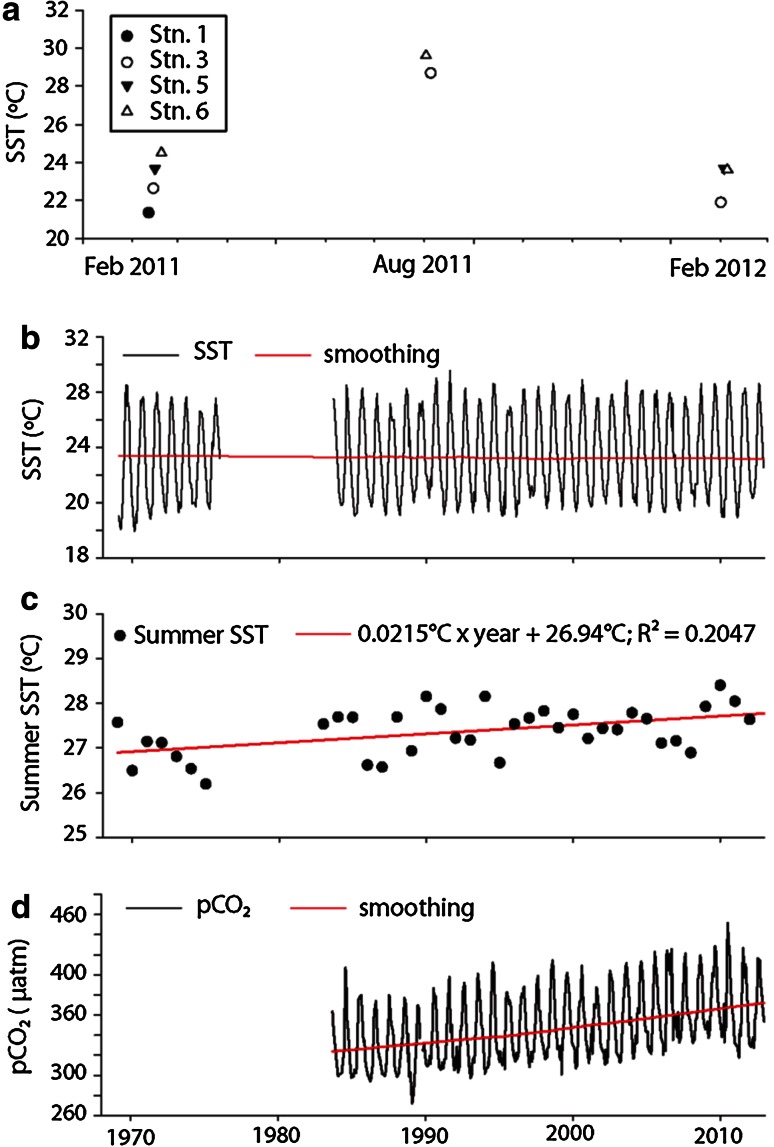
Sea surface temperature and pCO_2_, **a** Sea surface temperature at recent survey stations at time of sampling, **b** time series sea surface temperature (*black line*) and long-term trend (*red line*) collected at Hydrostation S (32**°**N, 64°W, near recent Station 1), **c** Summer sea surface temperature (*black dots*) and long-term trend (*red line*) at Hydrostation S, **d** pCO_2_ (*black line*) and long-term trend (*red line*) at Hydrostation S. Smoothing in **b** and **d** performed using negative exponential model and 0.7 sampling proportion. Data for panels **b**–**d** available through the Bermuda Atlantic Time Series program (Bates et al. 2012; Bates et al. 2014)

SST at recent sampling stations ranged from 21.3 **°**C in February 2011 to 29.6 **°**C in August 2011 (Fig. 2a). At Hydrostation S, year-round SST has not risen significantly since 1969 (*r* ^2^ = 0.0003, *F*_1,489_ = 0.1382, *P* = 0.7102; Fig. 2b). However, summer SST at Hydrostation S has increased slightly and gradually since 1969 (*r* ^2^ = 0.2098, *F*_1,35_ = 10.5561, *P* = 0026; Fig. 2c). Similarly, pCO_2_ at this station has increased steadily since 1984 [*r* ^2^ = 0.1566, *F*_1,378_ = 71.1882, *P* < 0001; Fig. 2d; Bates et al. 2012, 2014]. We found no relationship between SST and Shannon H′ log_e_ diversity index among the samples for which we had both of these values (*N* = 5 recent sites, 12 historical sites; *r* ^2^ = 0.0049, *F*_1,15_ = 0.0745, *P* = 0.7887).

### Mobile macrofauna community structure and diversity: recent surveys

In August 2011 and February 2012, we detected a total of 23 distinct taxa: eight distinct amphipods, one copepod, three decapods, two brachyurans, one tanaid, one polychaete, one snail, two nudibranchs, one pycnogonid, and three fishes. Total abundance varied greatly between samples (Table 1), ranging from 738 individuals collected at Sta. 6 in February 2012 to 9,586 individuals collected at Sta. 3 that same month. The decapod *Latreutes fucorum* was consistently the most abundant mobile macrofauna species encountered. We detected six out of ten species thought to be endemic to the Sargasso Sea, denoted by an asterisk in Table 1.

**Table 1:** Abundance (individuals L^−1^) of mobile macrofauna associated with *Sargassum* at four sample stations of recent study

Organism		Stations Aug 2011				Stations Feb 2012			
		1	3	5	6	1	3	5	6
Annelida: Phyllodocida									
*Platyneries dumerilli*		–	0	0	0	0.6	0	0	0
Crustacea: Amphipoda									
*Ampithoe* sp. A		–	0	0	0	30.0	11.5	0.9	1.3
*Ampithoe* sp. B		–	0	0	0	3.6	5.9	0.8	1.2
*Ampithoe longimana*		–	0	0	0	0.1	0.6	0	0.2
*Biancolina *brassicacephala*		–	0.1	0.1	0	0	0	0	0
*Hyale*		–	0	0.9	0	3.1	1.4	0.1	0
*Deutella*		–	0	0.1	3.6	0	0.3	0	0.1
*Sunamphitoe pelagica		–	0	0	0	26.6	9.5	0.7	0
Unidentified amphipod		–	0.4	1.1	0.6	29.1	5.3	0.8	0.3
Crustacea: Copepoda									
Unidentified copepod		–	0	0.5	0.2	0.1	0	0	0
Crustacea: Decapoda									
*Hippolyte coerulescens*		–	1.8	5.4	14.9	5.2	1.5	0.9	0.3
*Latreutes fucorum		–	124.1	195.6	214.3	472.9	908.3	112.7	67.5
*Leander tenicornis*		–	8	11.7	9.6	7.8	9.4	3.8	1.1
*Planes minutus		–	3.7	10.9	2.1	0.4	1.5	0.9	0.7
*Portunus sayi*		–	2.9	23.3	17.7	0.1	0.3	0.1	0.1
Crustacea: Tanaidacea									
Tanaid		–	0	0	0.1	0	0	0	0
Arthropoda: Pycnogonida									
Pycnogonid		–	0	0	0	0.1	0	0	0
Mollusca: Caenogastropoda									
*Litiopa melanostoma		–	2.2	0.5	14.3	0	0	1.2	0.6
Mollusca: Nudibranchia									
*Corambe obscura*		–	0	0	0	2.8	1.5	0.2	0
*Scyllaea pelagica		–	0	0	0	8.0	1.8	0.1	0.5
Chordata: Teleostei									
*Histrio histrio*		–	0.2	0.5	0.1	0.5	0	0	0
*Diodon*		–	0	0	0.1	0	0	0	0
Other fish		–	0.9	0	0	0	0	0	0
Total no. of individuals sampled (standardized to 10L sample)		–	1,443	2,506	2,776	5,909	9,586	1,232	738
Number of taxffoley		–	10	12	12	17	14	13	12

Original sample displacement volume exceeded 10 L for each station. Asterisks denote possible *Sargassum* community endemics (Laffoley et al. 2011)

**Fig. 3:**
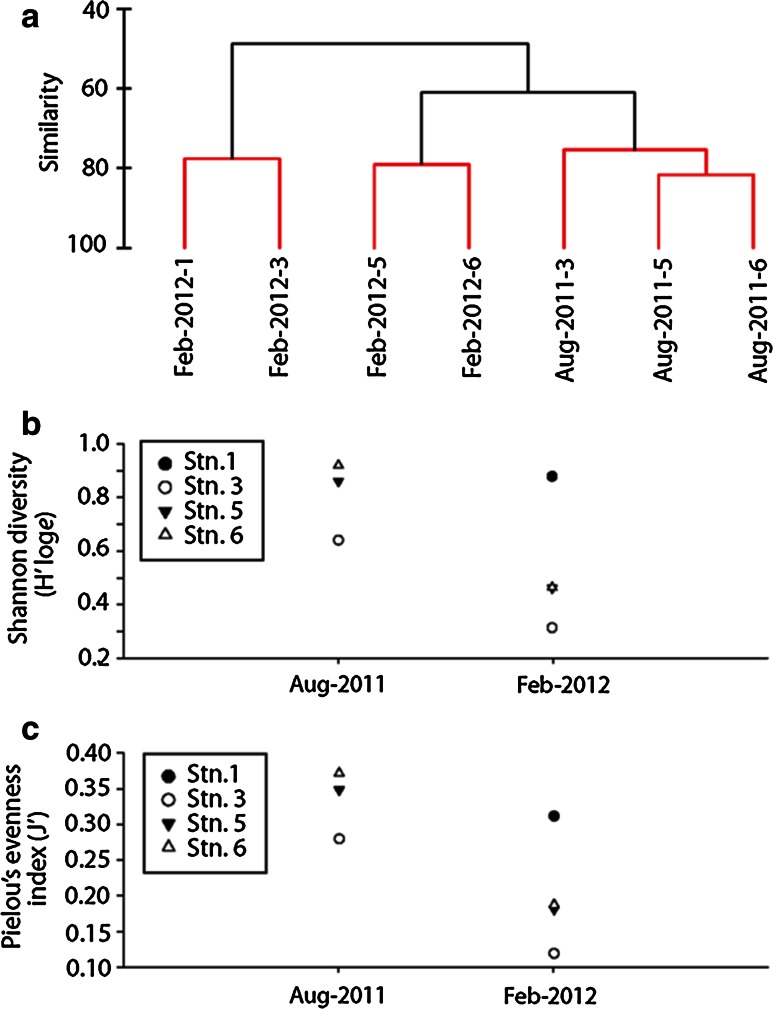
Mobile macrofauna community composition (MMCS) similarity, diversity, and evenness for Sargasso Sea stations examined, Aug 2011 and Feb 2012. **a** Cluster diagram showing percent similarity between samples. MMCS >48 % similar across sampling periods. *Red lines* denote statistically significant clustering according to the Simprof test, **b** Shannon diversity by recent cruise date. Station 1 (*black dot*) lies north of Sargasso Sea, 25°–29°N restricted block c) Pielou’s evenness index by recent cruise date. Station 1 (*black dot*) lies north of Sargasso Sea, 25°–29°N restricted block

For each recent cruise to the Sargasso Sea, MMCS did not cluster by station, and samples from Feb 2012 did not cluster by time period (Aug. 2011 and Feb. 2012; Table 1; ANOSIM Global *R* = 0.296, *P* = 17.1 %, Fig. 3a). For recent samples in the 25°–29°N restricted block (from Stations 3, 5, and 6), diversity and evenness at each station differed between the two cruises (diversity: Mann–Whitney U test *U* = 9, *N*_1_ = 3, *N*_2_ = 3, *P* = 0.05; evenness: Mann–Whitney *U* = 9, *N*_1_ = 3, *N*_2_ = 3, *P* = 0.05). Shannon *H*′ log_e_ diversity index averaged 0.8 ± 0.2 SD for the samples collected in Aug 2011 and 0.4 ± 0.09 SD in Feb 2012 (Fig. 3b). Pielou’s *J*′ evenness averaged 0.3 ± 0.05 SD in Aug 2011, and 0.2 ± 0.04 SD in and Feb 2012 (Fig. 3c) for those sites. Station 1 was not included in these diversity and evenness summaries because it lies north of the 25°–29°N block.

### Mobile macrofauna: comparison of historical and recent samples from the restricted block

**Fig. 4:**
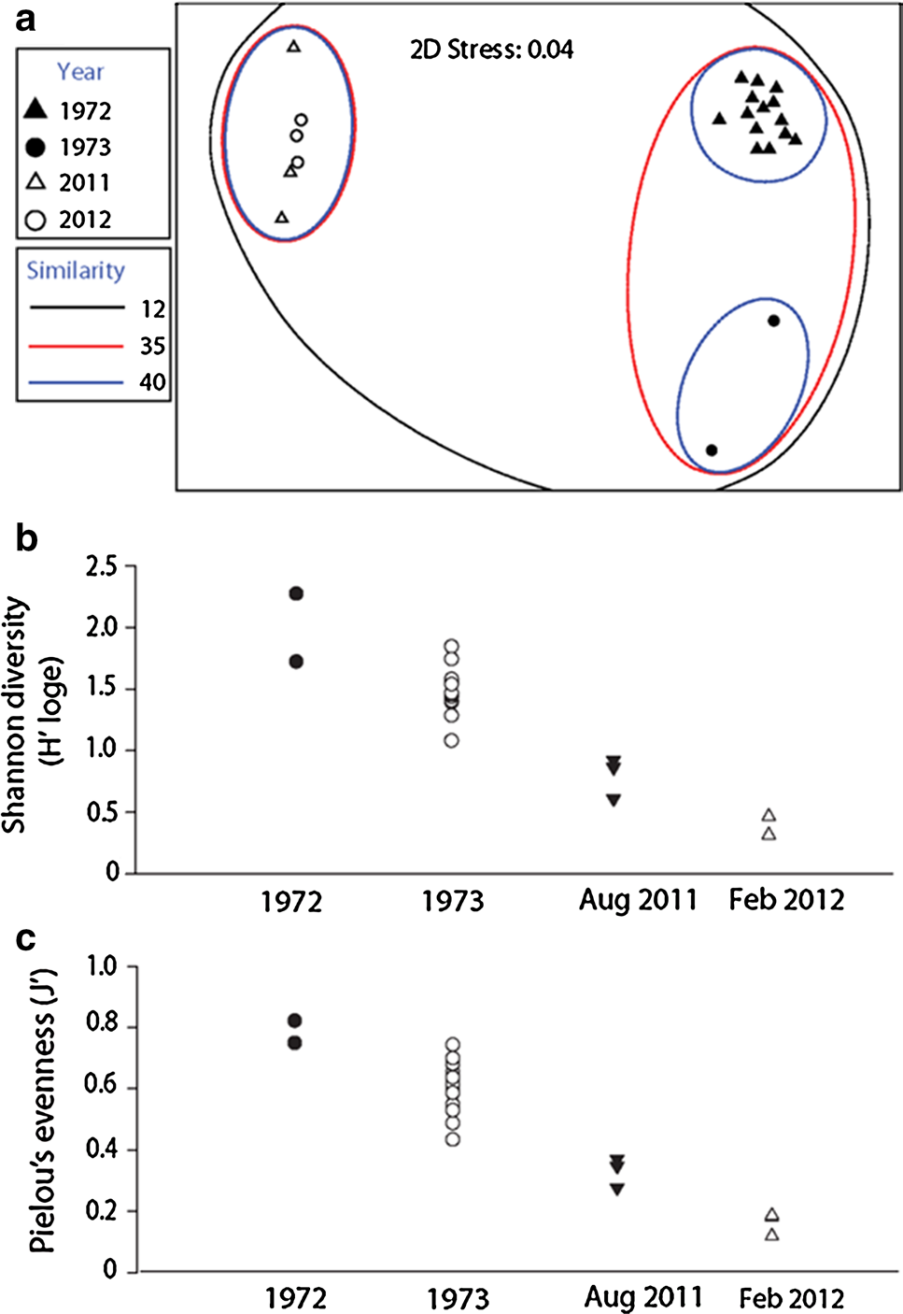
Recent and historical (Butler et al. 1983) samples of mobile fauna associated with *Sargassum* in Sargasso Sea, between 25° and 29°N (“restricted block” identified by *white triangles* in Fig. 1). **a** Non-metric multi-dimensional scaling plot of mobile fauna communities, with similarity cluster overlays. All diversity (**b**) and evenness (**c**) groups significantly different from each other based on pairwise Mann–Whitney *U* tests

Mobile macrofauna community structures documented in the restricted block in 2011 and 2012 were unlike those previously documented by historical cruises to the same area (ANOSIM Global *R* = 0.917, *P* = 0.01 %; Recent samples were <13 % similar to any of these historical samples; SIMPROF *Pi* = 14.09, *P* = 0.1 %; Fig. 4a). Diversity and evenness were significantly lower in Aug 2011 and Feb 2012 compared with the same measures from samples collected in 1972 and 1973 (diversity: Kruskal–Wallis *H*_3_ = 14.8, *P* = 0.002; evenness: Kruskal–Wallis *H*_3_ = 15.5, *P* = 0.0014; Fig. 4b, c, all groups were significantly different as determined by post hoc Mann–Whitney tests, with successive reductions between each sampling period). A measure of variability, the Multivariate Dispersion Index (MDI), was not significantly different for recent and historical samples from the restricted block (1.07 vs 1.00, respectively). Three flatworms, three nudibranchs, one isopod, two copepods, two decapods, and two pycnogonids were recorded in historical samples from the restricted block, but not in recent samples. By contrast, recent samples recorded seven amphipods and one tanaid that were not identified in historical samples in this area. However, this result could stem in part from improved taxonomic resolution of small crustaceans that did not appear to be present in historical samples. Because recent and historical dipnet collections of fishes could have yielded a biased subsample of the *Sargassum* community (Moser et al. 1998), we do not interpret the presence/absence of fishes.

### Mobile macrofauna: comparison of historical and recent samples from all latitudes and ecological groups

**Fig. 5:**
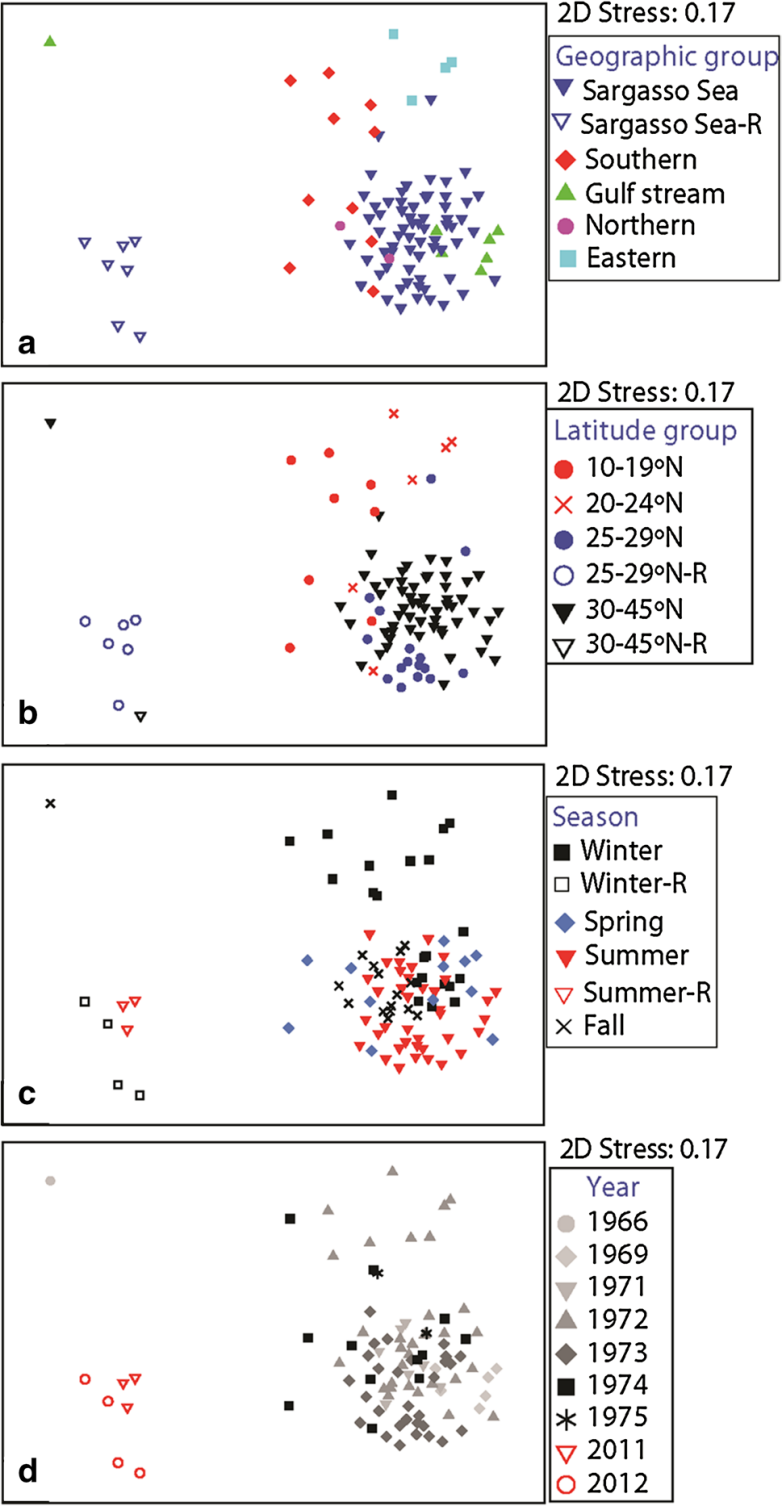
Multi-dimensional scaling plots of *Sargassum*-associated mobile macrofauna community structures documented in August 2011 and February 2012 (*open shapes*, legends designated with an “R”) compared with communities documented in historical samples collected between 1966 and 1975 (Weis 1968; Fine 1970; Butler et al. 1983), all geographic and latitude groups (both *orange and* *white triangles* in Fig. 1), labeled by **a** geographic group, **b** latitude group, **c** season, and **d** year

Recently sampled MMCS were also unlike any sample from the broader historical dataset, including those from samples from as far south as the Caribbean (orange and white triangles in Fig. 1; ANOSIM Global *R* = 0.606, *P* = 0.001 %). Recent MMCS were <19 % similar to any single historical sample MMCS (SIMPROF *Pi* = 6.9, *P* = 0.1 %), and <15 % similar to any historical sample in the Sargasso Sea (SIMPROF *Pi* = 5.69, *P* = 0.1 %; Fig. 5). The MMCS at historical stations in southern latitudes and those from recent stations were significantly different and did not indicate a northward latitudinal shift of species assemblages. Seasonal cyclicity in MMCS was once evident at Sta. S based on historical samples from 1972 and 1973, and in both those years communities returned to approximately the starting community compositions by the end of the year (Online Resource 2 panel a; RELATE test for cyclicity *ρ* = 0.23, *P* = 0.01 %). Our recent sample from Station 1 near Hydrostation S was far outside the range of historical variability at Hydrostation S (Online Resource 2 panel b).

### Epibiota percent cover

Each aliquot of *Sargassum* was identified, >95 % being *S. natans*. Six sessile taxa were documented growing attached to *Sargassum*. A total of 69.3 ± 22.9 % SD (*N* = 333 subsamples) of the plant in Feb 2011 and 78.5 ± 18.1 % SD (*N* = 335 subsamples) of the plant in February 2012 was free of epibiont coverage (Table 2). Although average coverage by all epibiont taxa was lower in Feb 2012 than Feb 2011, there was no significant difference between these groups (all pairwise tests *P* > 0.2). For each aliquot, we recorded the presence or absence of each growth zone (A–F of Ryland 1974). All growth stages were represented in *Sargassum* from all sampling stations. All growth zones were equivalently present during Feb 2011 (ANOVA, *F*_(5,25)_ = 2.366, *P* > 0.08). During Feb 2012 (ANOVA *F*_(5,30)_ = 2.797, *P* < 0.035), growth zone E was significantly less common among aliquots than growth zones B and F (Wilcoxon Signed Rank *P* = 0.03 for each comparison).

**Table 2:** Total coverage of epibiota growth on *Sargassum* in Feb 2011 (1) and Feb 2012 (3), and percent cover ± standard deviation for *Sargassum* growth zones and plant parts averaged across sampling periods

Epibiota	Total % cover ± SD	Zone mean % cover ± SD		Plant part mean % cover ± SD	
Crustose coralline algae (*Melabersia*)	(1) 9.5 ± 13.4 (3) 7.4 ± 12.5	A	4.2	Bladder	6.7
		B	8.3	Leaf	9.4
		C	41.7	Stem	9.2
		D	10.8		
		E	8.5		
		F	8.1		
Macroalgae (*Dictyota*)	(1) 0.0 ± 0.5 (3) 0.0 ± 0.0	A	0.0	Bladder	0.0
		B	0.0	Leaf	0.0
		C	0.0	Stem	0.1
		D	0.0		
		E	0.0		
		F	0.1		
Bryozoa (*Membranipora* spp.)	(1) 10.7 ± 17.0 (3) 5.5 ± 13.4	A	3.8	Bladder	6.2
		B	5.0	Leaf	5.6
		C	26.6	Stem	1.24
		D	8.8		
		E	11.0		
		F	14.2		
Cnidaria (*Clytia noliformis, Obelia dichotoma* and *Plumularia* sp.)	(1) 9.1 ± 8.5 (3) 7.3 ± 7.7	A	10.9	Bladder	4.7
		B	9.4	Leaf	9.0
		C	12.4	Stem	10.9
		D	8.3		
		E	7.3		
		F	5.6		
Nereididae (*Spirorbus*)	(1) 0.7 ± 2.4 (3) 0.6 ± 2.1	A	0.4	Bladder	0.6
		B	0.4	Leaf	0.3
		C	0.6	Stem	1.0
		D	0.5		
		E	0.7		
		F	0.3		
Peduculata (*Lepas pectinata*)	(1) 0.7 ± 2.3 (3) 0.7 ± 2.8	A	1.1	Bladder	0.3
		B	1.0	Leaf	0.4
		C	1.3	Stem	1.5
		D	0.5		
		E	0.8		
		F	0.4		
No coverage	(1) 69.3 ± 22.9 (3) 78.5 ± 18.1				
		A	79.6	Bladder	81.6
		B	75.9	Leaf	75.3
		C	17.4	Stem	64.9
		D	71.1		
		E	71.7		
		F	70.4		

### Trophic groups: historical and recent comparison

**Fig. 6:**
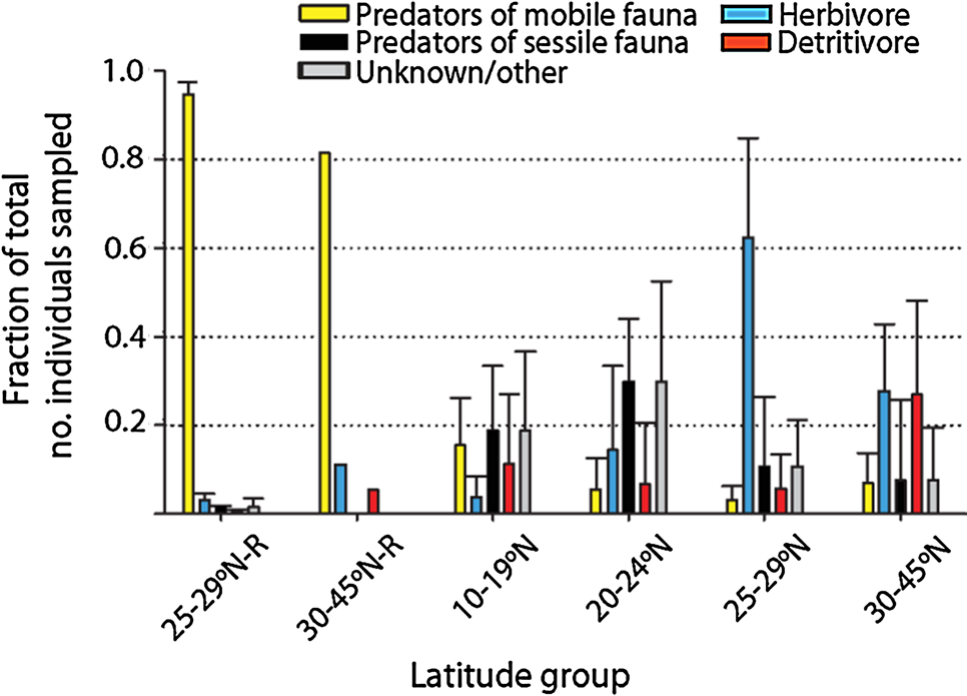
Trophic groups of mobile macrofauna associated with *Sargassum* in recent and historical samples. Relative fraction of all individuals in each sample represented by each trophic group, an average of all samples in each latitude block ± standard deviation *error bars*. Groups specified by an R represent recent surveys. The other/unknown category includes parasites and taxa for which diets were unknown. Trophic category designations and citations in Online Resource 3

Recent samples were dominated by mobile macrofauna that are predators of other mobile macrofauna (Fig. 6; Online Resource 3), a pattern dominated by relatively high abundance of the decapod crustacean *Latreutes fucorum* compared to the other organisms of known trophic group. By contrast, historical sites to the south had a higher relative representation of epibiont predators and detritivores, and historical sites to the north had a higher relative representation of herbivores and detritivores. We were unable to define the diet of *Litiopa melanostoma*, a gastropod that was common in historical datasets.

## Discussion

### Oceanographic conditions (SST and pCO_2_)

The Sargasso Sea has undergone seasonal and long-term changes in oceanographic characteristics that might have influenced floating *Sargassum* and its rafting communities. In the past 30–40 years, the summer SST and mean pCO_2_ have increased steadily at Hydrostation S near Bermuda, in close proximity to Station 1 (Fig. 2c, d; Bates et al. 1998, 2012, 2014; Steinberg et al. 2012). Over time, there has also been a northward shift in a key isotherm (22.5 °C) in the Sargasso Sea (Friedland et al. 2007) and a slight increase in wind speed and wave height during extreme weather events (Young et al. 2011). If the trends experienced at Hydrostation S are representative of those experienced by the broader Sargasso Sea, then our sampling periods took place during two variable years in the Sargasso Sea’s temperature history. Our survey in February 2011 took place during the second coolest February since 1970. The survey in August 2011 took place during a period of average summer SST. Finally, the survey in February 2012 took place during one of the top 6 warmest February months since 1970. All of our samples were from waters 21 °C or warmer.

### Mobile macrofauna community structure and diversity: recent surveys

Our result suggested a lack of site-specific community structure at the time of sampling, as well as inconsistent clustering by cruises in 2011 and 2012. We documented variation in mobile macrofauna assemblages and declines in diversity, evenness, and abundance within a six-month time period between August 2011 and February 2012 (Fig. 3b, c; Table 1). More than half of the animal groups documented in one of these sampling periods were not documented in the other (Table 1). These differences can be attributed to changes in the amphipod community, the addition of two nudibranchs in 2012, and isolated counts of taxa documented in low numbers (Table 1).

### Mobile macrofauna: comparison of historical and recent samples from the restricted block

As hypothesized, recently sampled mobile macrofauna communities associated with *Sargassum* in the restricted block (Sargasso Sea 25°–29°N) deviated significantly from those communities documented historically in the same area. However, the MMCS were variable across both recent and historical sample groups, exhibiting statistically similar inter-sample variability over the 1972–1973 and 2011–2012 time periods, respectively (similar MDI, 35 vs 40 % similarity, respectively; Fig. 4a). Significant declines in diversity and evenness were also documented between 1972 and 1973 (Fig. 4b, c), similar in magnitude to the declines from 2011 to 2012.

### Mobile macrofauna: comparison of historical and recent samples in all latitudes and ecological groups

At the basin-wide scale, the *Sargassum* mobile macrofauna community appears to have exhibited a fair degree of variability over time and space. Recent MMCS were unlike any historical communities we analyzed, whether compared to sites of the same latitude and geographic region (Fig. 4a), or the broader historical dataset including sites across the Sargasso Sea, Gulf Stream, south of the subtropical convergence zone, and in northern latitudes (Fig. 5). Fine (1970) observed considerable changes in species dominance between samples and concluded each raft reflected a separate community, with separate colonization events. In the late 1960s, *Sargassum* in the Gulf Stream and Sargasso Sea supported similar mobile macrofauna communities (Fine 1970), a pattern that was not observed 10 years later (Stoner and Greening 1984). Samples from these two historic collections shared only two of their respective top five most abundant species. Each recent sampling period reported here shared two of the top five most abundant taxa with those documented by Stoner and Greening (1984), but exhibited no overlap with Fine’s samples from the Sargasso Sea (Fine 1970). The *Sargassum* mobile macrofauna communities sampled near Hydrostation S exhibited seasonal cyclicity in the 1970s and appeared to return to a starting community composition each year (Online Resource 2, panel a). By contrast, communities we sampled near this site in 2012 fell very far outside this cyclical pattern (Online Resource 2, panel b). Clarkin et al. (2012) concluded that organisms inhabiting floating habitats of the Irish coast represented a random subsample of the broader rafting community there. More refined sampling is required before we can evaluate the impact of recruitment on spatiotemporal patterns of rafting animal MMCS and diversity in the Sargasso Sea.

### Epibiota percent cover

Our samples recorded low coverage by epibionts, including calcifying bryozoans, *Spirorbus*, *Lepas*, and crustose coralline algae, but also cnidarians (primarily hydroids), which are non-calcifying. Bryozoans, crustose coralline algae, and cnidarians each exhibited average coverage of approximately 10 % or less. *Spirorbus* and *Lepas* demonstrated much lower coverage (>1 % coverage; Table 2). Although others have found a positive correlation between *Sargassum* age and epibiont coverage (Ryland 1974; Stoner and Greening 1984), this relationship does not appear to explain our results. In February 2012, epibiont coverage was low (total avg. coverage 21 %) despite an abundance of an advanced algal growth stage, Stage F, which indicates senescent seaweed. Our finding of *Sargassum* bearing the full range of growth stages but low epibiont coverage conflicts with previous reports (Ryland 1974; Stoner and Greening 1984). Additionally, our findings cannot be explained by variation in SST. Epibiont coverages in February 2011 and February 2012 were equally low, even though the Sargasso Sea was especially cool and warm during these times, respectively.

Although differences in methods do not allow for direct statistical comparison, coverage by calcifying bryozoans in our samples was low compared to coverage in samples quantified from the Sargasso Sea in the late 1970s (Niermann 1986). Niermann recorded a substantial portion of bladders with greater than 75 % surface area covered by bryozoans, more so north of the 21 °C isotherm than to the south (22–38 % of bladders with >75 % coverage to the north vs. 4–10 % of bladders with >75 % coverage to the south; Niermann 1986). By contrast, none of the bladders in our samples had such extensive bryozoan growth, or even coverage by all epibiont forms combined. All of our samples came from waters warmer than 21 °C and had considerably less coverage than those collected from southern sites in 1979 (Niermann 1986). We are unable to compare our values for *Spirorbus*, *Lepas*, and hydroids to other historical accounts, which were either qualitative assessments of “rare” to “common,” or presented as presence–absence data (Weis 1968; Butler et al. 1983; Calder 1995).

### Trophic groups: historical and recent comparison

The feeding ecology of Sargasso Sea macrofaunal communities has been generalized as being dominated by suspension feeders, then grazers and browsers, followed by predators, and detritus feeders (Thiel and Gutow 2005b). Our observations differed from this simplified pattern. We observed low coverage by sessile filter feeders, an especially high fraction of the predator *Latreutes fucorum*, loss of several soft-bodied predators of sessile fauna, and low numbers of grazers and detritivores (Fig. 6). As found previously, we documented macrofauna living in *Sargassum* that share food sources; most of the species appear to be generalists with many food sources (Butler et al. 1983). While preliminary, our observations might reflect a combination of bottom-up and top-down influences on mid-level consumers. Half of the mobile macrofauna present in historic Sargasso Sea samples but absent from our samples (Table 3) are soft-bodied nudibranchs and flatworms that prey on sessile fauna (epibiota). While flatworms can be generalist predators and scavengers (Jennings 1957), many nudibranchs have species-specific feeding morphologies and ecologies [as with nudibranchs feeding on kelp epibionts (Lambert 1991)]. The epibiont community we observed recently might have been insufficient to support a diversity of predators. Concurrently, the disproportionally high abundance of *Latreutes fucorum*, which can prey on mobile macrofauna (Butler et al. 1983), could suggest top-down control of mid-level consumers, herbivores, and/or detritivores. Amphipods can exert indirect impacts on community composition and invertebrate species richness through selective grazing activity and modification of their macroalgal habitat (Duffy and Hay 2000). However, we do not know how the changes in amphipod species composition might have impacted our observations.

**Table 3:** Presence (+) or absence (−) of mobile macrofauna associated with *Sargassum* in recent and historical samples in the restricted block (Sargasso Sea, 25°–29°N) and historical samples outside this restricted block

	Recent: Sargasso Sea 25°–29°N	Historic: Sargasso Sea 25°–29°N	Historic: outside Sargasso Sea 25°–29°N
Annelida: Polychaeta			
Polychaeta	−	+	+
Spionid worm	−	−	+
Annelida: Phyllodocida			
*Myrianida*	−	−	+
*Platynereis dumerilii*	+	+	+
Arthropoda			
Ostracod	−	−	+
Crustacea: Amphipoda			
Amphipod unidentified	+	−	−
*Ampithoe A*	**+**	−	−
*Ampithoe B*	**+**	−	−
*Ampithoe longimana*	**+**	−	−
*Biancolina spp.*	+	+	+
*Deutella incerta*	+	−	−
*Gammarus*	−	−	+
*Hyale*	+	−	−
*Luconacia*	+	−	−
*Sunamphitoe pelagica*	+	+	+
Crustacea: Copepoda			
Copepod unidentified	+	+	−
***Dactylopusia tisboides***	−	+	−
***Scutellidium longicauda***	−	+	−
Crustacea: Decapoda			
***Hemiaegina minuta***	−	+	+
*Hippolyte coerulescens*	+	+	+
*Latreutes fucorum*	+	+	+
*Leander tenuicornis*	+	+	+
*Planes minutus*	+	+	+
*Portunus sayi*	+	+	+
*Tozeuma* *carolinense*	−	−	+
Shrimp	−	+	+
Crustacea: Isopoda			
***Carpias*** ***minutus***	−	+	+
*Cirolana*	−	−	+
*Grapsicepon*	−	−	+
*Idotea metallica*	−	−	+
*Probopyrinella latreuticola*	−	−	+
Isopod unidentified	−	+	+
Parasitic isopod unidentified	−	+	+
Crustacea: Tanaidacea			
Tanaid	+	−	+
Arthropoda: Pycnogonida			
***Anoplodactylus petiolatus***	−	+	+
***Endeis spinosa***	−	+	+
Pycnagonid unidentified	+	−	−
Mollusca: Caenogastropoda			
*Bittium*	−	−	+
*Janthina janthina*	−	−	+
*Litiopa melanostoma*	+	+	+
*Rissoa*	−	−	+
Gastropod unidentified	−	−	+
Mollusca: Nudibranchia			
*Corambe obscura*	+	−	+
*Cuthona*	−	−	+
***Doto pygmaea***	−	+	+
*Fiona pinnata*	−	−	+
*Scyllaea pelagica*	+	+	+
***Spurilla neapolitana***	−	+	+
Nudibranch B	−	−	+
**Nudibranch D**	−	+	+
Nudibranch unidentified	−	−	+
Platyhelminthes			
***Acerotisa notulata***	−	+	+
* ***Chatziplana grubei***	−	+	+
***Gnesioceros sargassicola***	−	+	+
*Polycladus*	−	−	+
Flatworm unidentified	−	−	+
Xenacoelomorpha: Acoela			
*Heterochaerus sargassi*	−	−	+
Nemertea			
*Nematoda*	−	−	+
Echinodermata			
Ophioridae	−	−	+
Chordata: Thaliacea			
*Salpa*	−	−	+
Chordata: Teleostei			
*Balistes*	−	−	+
*Caranx*	−	−	+
*Diodon*	+	−	−
*Histrio histrio*	+	+	+
*Stephanolepis hispidus*	−	−	+
*Syngnathus typhle*	−	−	+
Fish unidentified	+	−	−
Sipuncula			
Sipunculid larva	−	−	+

Possible losses from the restricted block in bold. An asterisk denotes possible loss of Sargasso Sea endemic from this region

### Conclusions

*Sargassum* features that are visible by satellite appear to circulate seasonally, and occasionally exhibit high inter-annual variation in estimated biomass and circulation patterns. Typically, this habitat appears to be advected from a source in the Gulf of Mexico each spring, through the Florida Strait to the Gulf Stream in the summer, and then circulates with prevailing currents to pass northeast of the Bahamas in February (Gower and King 2011). However, the latitude at which *Sargassum* features can be found during each calendar month varies (Gower and King 2011). Given the very different circulation histories of the *Sargassum* habitats sampled in 2011 and 2012, we were not surprised to see variability in macrofauna communities over time and space. In these years, the distribution, abundance, and/or circulation patterns of *Sargassum* features in the Atlantic were anomalous compared to the 2003–2010 period and exceed variability of those factors in living human memory (Gower and King 2011; Gower et al. 2013; Smetacek and Zingone 2013). In 2010, *Sargassum* biomass was very low, perhaps because of adverse effects caused by pollution from the Deepwater Horizon oil spill and cleanup efforts in the Gulf of Mexico (Powers et al. 2013). In 2011, the major source of *Sargassum* was a region offshore of the Amazon rather than the Gulf Stream and unprecedented masses washed ashore in the Caribbean or drifted eastward at low latitudes all the way to Africa (Gower et al. 2013). The majority of that *Sargassum* biomass never appeared to reach the Sargasso Sea region, having never met with the Gulf Stream and circulated northward accordingly (Gower et al. 2013). By the time of our second sampling (August, 2011), high *Sargassum* biomass was situated in the Caribbean, but very little was visible in our study area to the north (Gower et al. 2013). These seasonal and yearly differences in advection of the *Sargassum* would contribute to variation in recruitment source populations and temperatures experienced by the inhabiting macrofauna and epibiota (Butler et al. 1983). Offshore rafting communities can be depauperate compared to their coastal counterparts (Ingólfsson 1995). As such, conclusions drawn from one time or geographic area in the Sargasso Sea might not necessarily offer valid comparisons for another region or time (Riemann et al. 2011). These characteristics might confound our ability to evaluate changes in *Sargassum* macrofauna communities over time. Therefore, large-scale oceanographic circulation could have significant implications for understanding *Sargassum* community variation in both long- and short-term cycles.

From theoretical predictions to empirical evidence, surface warming and increased pCO_2_ are associated with a host of changes capable of influencing marine populations at a broad scale (Friedland et al. 2007; Pinsky et al. 2013), especially rafting communities (Macreadie et al. 2011). Contrary to our original hypothesis, we did not observe a northward shift of mobile macrofauna communities. On the whole, the MMCS for recent samples from the 25°–29°N latitude block were significantly different from MMCS of historic samples from farther south. Range shifts in response to warming are not uniform in their magnitude and direction (Stefansdottir et al. 2010; Pinsky et al. 2013), an aspect that confounds our ability to detect distributional shifts of entire communities. Although our samples are limited, SST alone does not appear to be a primary variable influencing diversity. We found no correlation between Shannon H′ and SST. Likewise, we did not see a loss of mobile macrofauna biased toward calcifying forms. When interpreted together with the broader literature about climate change impacts on marine life (Friedland et al. 2007; Pinsky et al. 2013), our results support the idea that different species, and entire animal communities, can vary in their sensitivity to pH (Martin et al. 2008; Rodolfo-Metalpa et al. 2010) and response to ocean warming (Stefansdottir et al. 2010; Pinsky et al. 2013).

However, it is possible that increasingly acidic conditions of the Sargasso Sea have contributed to our observed reduction in coverage by epibionts, especially bryozoans such as *Membranipora*, which is lightly calcified (Banta et al. 1995). Calcifying animals might experience difficulty building exoskeletons and shells, and attaching to *Sargassum* in lower pH sea water, as seen in other studies (Martin et al. 2008). *Sargassum* with low epibiont coverage, and its associates, might experience increased UV exposure because of reduced overgrowth and reduced localized supply of nutrients from epibiont waste excretion (Thiel and Gutow 2005b; Rothäusler et al. 2012). If low coverage also contributes to reduced sinking rates, because clumps do not sink from being overgrown and weighed down (Rothäusler et al. 2012), then the rate of food supply of *Sargassum* to the deep sea would also be reduced. Fish and invertebrate scavengers in the Sargasso Sea’s deep waters (> 5,000 m) consume fallen *Sargassum* and epibiota and could be impacted by changes in surface *Sargassum* communities (Fleury and Drazen 2013).

The diversity and species composition of macrofauna communities associated with *Sargassum* might be inherently unstable. Diversity, evenness, and MMCS of rafting communities in the Sargasso Sea showed little consistency over time or space. In some marine communities, stability has been attributed to a lack of local stressors (e.g., Elahi et al. 2013), a trait that does not apply to the Sargasso Sea. Local and basin-wide stressors include increasing wind disturbances, SST, acidification (Bates et al. 1998, 2012, 2014; Friedland et al. 2007; Steinberg et al. 2012), large shifts in phytoplankton communities and nutrient cycling (Morán et al. 2010; Steinberg et al. 2012), widening geographic range of tropical species (Piontkovski and Castellani 2009), and increasing ship traffic (Trott et al. 2010). Fisheries have significantly altered the abundance of fishes and whales in the Sargasso Sea (Hallett, 2011). The same currents that transport *Sargassum* and larval recruits might also serve to concentrate pollutants known to occur in the region (Trott et al. 2010), including hydrocarbons (Butler et al. 1983), heavy metals (Johnson and Braman 1975), plastics (Carpenter and Smith 1972), and oil spill cleanup chemicals (Powers et al. 2013). By 2050, the Sargasso Sea is predicted to experience a moderate increase in species turnover rate due to range shifts in response to warming [Cheung et al. 2009; “turnover rate” is considered “The number of species eliminated and replaced per unit time” as defined by MacArthur and Wilson (1967)]. While macrofauna communities sampled throughout the Sargasso Sea in 2011 and 2012 had deviated significantly from those inhabiting that region in the 1970s, only long-term sampling can reveal whether they might (1) return to original assemblages, (2) sustain alternate community compositions, or (3) continue to undergo major change. Regular monitoring of the *Sargassum* community, at minimum every decade, is necessary to determine whether our results signal a long-term decline in this ecosystem, or low points in naturally variable diversity.

## Electronic supplementary material

Below is the link to the electronic supplementary material.

Supplementary material 1 (PDF 160 kb)

Supplementary material 2 (PDF 126 kb)

Supplementary material 3 (PDF 203 kb)
