# Supplementary material for: Pelagic Sargassum community change over a 40-year period: temporal and spatial variability
Source: Mar Biol. 2014 Sep 14;161(12):2735–51. doi: 10.1007/s00227-014-2539-y (PMC4231207; doi:10.1007/s00227-014-2539-y)
Supplement: Supplementary file 1 — Supplementary material 1 (PDF 160 kb) [file 227_2014_2539_MOESM1_ESM.pdf]

Supplementary Table 1. Stations analyzed in this study, along with site location and data source. Note sites <70 ml DV were not included in analyses.

Pelagic *Sargassum* community change over a 40-year period: temporal and spatial variability. *Marine Biology* C.

L. Huffard[*], S. von Thun, A. D. Sherman, K. Sealey, K. L. Smith, Jr.
*Corresponding author: Monterey Bay Aquarium Research Institute, 7700 Sandholdt Rd, Moss Landing, CA 95039; chuffard@mbari.org, phone: +1-831-775-1839, fax: (831) 775-1620

Cruise	Year	Season	Sample code	Latitude	Longitude	Data source
Weis	1966	4	Weis	34.2	-75.82	Weis, 1968
Fine	1969	3	Fine 1	34.35	-75.6	Fine, 1970; Butler et al, 1983
Fine	1969	3	Fine 1a	33.81	-73.54	Fine, 1970; Butler et al, 1983
Fine	1969	3	Fine 2	33.93	-74.45	Fine, 1970; Butler et al, 1983
Fine	1969	3	Fine 3	33.53	-72.62	Fine, 1970; Butler et al, 1983
Fine	1969	3	Fine 4	33.43	-71.93	Fine, 1970; Butler et al, 1983
Fine	1969	3	Fine 5	33.25	-71.02	Fine, 1970; Butler et al, 1983
Hysdostation S	1971	3	HS-346	32.17	-64.5	Butler et al., 1983
Hysdostation S	1971	3	HS-347	32.17	-64.5	Butler et al., 1983
Hysdostation S	1971	3	HS-348	32.17	-64.5	Butler et al., 1983
Hysdostation S	1971	3	HS-349	32.17	-64.5	Butler et al., 1983
Hysdostation S	1971	4	HS-353	32.17	-64.5	Butler et al., 1983
Hysdostation S	1971	4	HS-354	32.17	-64.5	Butler et al., 1983
Hysdostation S	1971	4	HS-355	32.17	-64.5	Butler et al., 1983
Hysdostation S	1972	1	HS-356	32.17	-64.5	Butler et al., 1983
Hysdostation S	1972	1	HS-373	32.17	-64.5	Butler et al., 1983
Hysdostation S	1972	1	HS-374	32.17	-64.5	Butler et al., 1983
Sir Horace Lamb	1972	1	SHL-1	29.17	-63.75	Butler et al., 1983
Sir Horace Lamb	1972	1	SHL-2	28.22	-63.5	Butler et al., 1983
Sir Horace Lamb	1972	1	SHL-3	24.72	-62.48	Butler et al., 1983
Sir Horace Lamb	1972	1	SLH-4	21.83	-61.8	Butler et al., 1983
Sir Horace Lamb	1972	1	SHL-5	23	-62.77	Butler et al., 1983
Sir Horace Lamb	1972	1	SLH-6	21.92	-61.55	Butler et al., 1983
Sir Horace Lamb	1972	1	SLH-7	18.24	-62.88	Butler et al., 1983
Sir Horace Lamb	1972	1	SLH-8	18.11	-62.75	Butler et al., 1983
Sir Horace Lamb	1972	1	SLH-9	18.33	-60.78	Butler et al., 1983
Hysdostation S	1972	2	HS-358	32.17	-64.5	Butler et al., 1983
Hysdostation S	1972	2	HS-360	32.17	-64.5	Butler et al., 1983
Hysdostation S	1972	2	HS-361	32.17	-64.5	Butler et al., 1983
Hysdostation S	1972	3	HS-364	32.17	-64.5	Butler et al., 1983
Hysdostation S	1972	3	HS-365	32.17	-64.5	Butler et al., 1983
Hysdostation S	1972	3	HS-366	32.17	-64.5	Butler et al., 1983
Westward-72	1972	3	W-1	32.67	-63	Butler et al., 1983
Westward-72	1972	3	W-2	33.42	-59.75	Butler et al., 1983
Westward-72	1972	3	W-3	33.58	-57.33	Butler et al., 1983

Westward-72	1972	3	W-4	38.42	-50.25	Butler et al., 1983
Westward-72	1972	3	W-5	35.6	-45.05	Butler et al., 1983
Halifax	1972	4	D-22	44.5	-62.5	Butler et al., 1983
Halifax	1972	4	D-23	44.5	-62.5	Butler et al., 1983
Hysdostation S	1972	4	HS-367	32.17	-64.5	Butler et al., 1983
Hysdostation S	1972	4	HS-368	32.17	-64.5	Butler et al., 1983
Hysdostation S	1972	4	HS-369	32.17	-64.5	Butler et al., 1983
Hysdostation S	1972	4	HS-370	32.17	-64.5	Butler et al., 1983
Hysdostation S	1972	4	HS-371	32.17	-64.5	Butler et al., 1983
Hysdostation S	1972	4	HS-372	32.17	-64.5	Butler et al., 1983
Hysdostation S	1973	1	HS-375	32.17	-64.5	Butler et al., 1983
Hysdostation S	1973	1	HS-376	32.17	-64.5	Butler et al., 1983
Hysdostation S	1973	1	HS-377	32.17	-64.5	Butler et al., 1983
Hysdostation S	1973	2	HS-378	32.17	-64.5	Butler et al., 1983
Hysdostation S	1973	2	HS-380	32.17	-64.5	Butler et al., 1983
Eastward	1973	3	E-22317	28.42	-71.38	Butler et al., 1983
Eastward	1973	3	E-22319	29.64	-69.7	Butler et al., 1983
Eastward	1973	3	E-22310	29.87	-69.43	Butler et al., 1983
Eastward	1973	3	E-22310A	29.87	-69.43	Butler et al., 1983
Eastward	1973	3	E-22312	27.93	-70.03	Butler et al., 1983
Eastward	1973	3	E-22313	27.5	-70.63	Butler et al., 1983
Eastward	1973	3	E-22321	28.52	-69.17	Butler et al., 1983
Eastward	1973	3	E-22324	29.82	-71.42	Butler et al., 1983
Eastward	1973	3	E-22309	30.53	-68.24	Butler et al., 1983
Eastward	1973	3	E-22327	32.43	-74.28	Butler et al., 1983
Eastward	1973	3	E-22328	32.77	-74.67	Butler et al., 1983
Hunt	1973	3	Hunt-A	27.07	-72.67	Butler et al., 1983
Hunt	1973	3	Hunt-B	26.18	-75.78	Butler et al., 1983
Hunt	1973	3	Hunt-C	27.12	-72.67	Butler et al., 1983
Hunt	1973	3	Hunt-1	28.97	-69.65	Butler et al., 1983
Hunt	1973	3	Hunt-1b	28.97	-69.65	Butler et al., 1983
Hunt	1973	3	Hunt-2	26.46	-69.33	Butler et al., 1983
Hysdostation S	1973	3	HS-382	32.17	-64.5	Butler et al., 1983
Hysdostation S	1973	3	HS-383	32.17	-64.5	Butler et al., 1983
Hysdostation S	1973	3	HS-385	32.17	-64.5	Butler et al., 1983
Hysdostation S	1973	4	HS-386	32.17	-64.5	Butler et al., 1983
Hysdostation S	1973	4	HS-387	32.17	-64.5	Butler et al., 1983
Hysdostation S	1973	4	HS-388	32.17	-64.5	Butler et al., 1983
Hysdostation S	1973	4	HS-389	32.17	-64.5	Butler et al., 1983
Hysdostation S	1974	1	HS-390	32.17	-64.5	Butler et al., 1983
Hysdostation S	1974	1	HS-391	32.17	-64.5	Butler et al., 1983
Hysdostation S	1974	1	HS-392	32.17	-64.5	Butler et al., 1983
Westward 1974	1974	1	W-25-Jan	17.33	-62.33	Butler et al., 1983
Westward 1974	1974	1	W-8-Feb	18	-67.5	Butler et al., 1983
Hysdostation S	1974	2	HS-393	32.17	-64.5	Butler et al., 1983

Hysdostation S	1974	2	HS-394	32.17	-64.5	Butler et al., 1983
Westward 1974	1974	2	W-8-Mar	16.65	-84.63	Butler et al., 1983
Westward 1974	1974	2	W-8 Mar-b	16.65	-84.78	Butler et al., 1983
Westward 1974	1974	2	W-8 Mar-c	16.67	-84.93	Butler et al., 1983
Westward 1974	1974	2	W-17 Mar	23.98	-84.48	Butler et al., 1983
Westward 1974	1974	2	W-24 Mar	24.18	-79.8	Butler et al., 1983
Hysdostation S	1975	1	HS-398	32.17	-64.5	Butler et al., 1983
Hysdostation S	1975	1	HS-399	32.17	-64.5	Butler et al., 1983
Smith	2011-2012	1	S-1	31.91	-64.04	This study
Smith	2011-2012	1,3	S-3	29.61	-66.54	This study
Smith	2011-2012	1,3	S-6	25.5	-71	This study
Smith	2011-2012	1,3	S-5	26.74	-69.71	This study
