# Supplementary material for: Pelagic Sargassum community change over a 40-year period: temporal and spatial variability
Source: Mar Biol. 2014 Sep 14;161(12):2735–51. doi: 10.1007/s00227-014-2539-y (PMC4231207; doi:10.1007/s00227-014-2539-y)
Supplement: Supplementary file 2 — Supplementary material 2 (PDF 126 kb) [file 227_2014_2539_MOESM2_ESM.pdf]

Pelagic *Sargassum* community change over a 40-year period: temporal and spatial variability. *Marine Biology* C.

L. Huffard*, S. von Thun, A. D. Sherman, K. Sealey, K. L. Smith, Jr.
*Corresponding author: Monterey Bay Aquarium Research Institute, 7700 Sandholdt Rd, Moss Landing, CA 95039; chuffard@mbari.org, phone: +1-831-775-1839, fax: (831) 775-1620

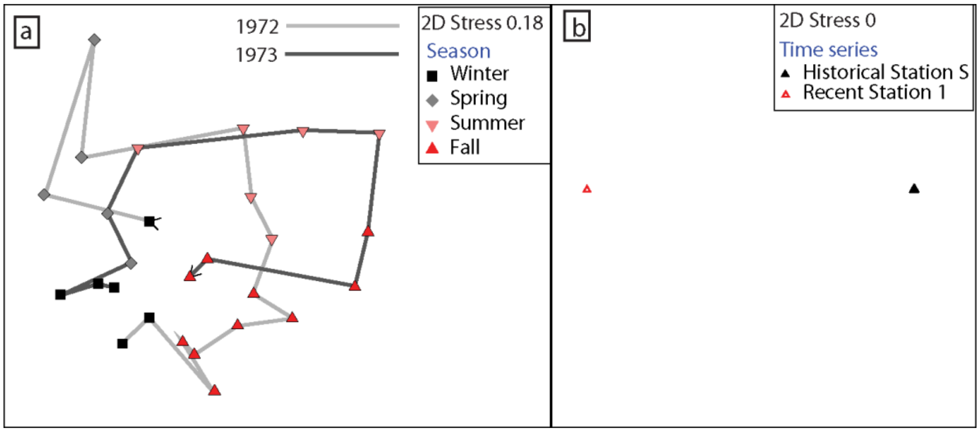

Fig. Supplementary figure 1 Non-metric multi-dimensional scaling (MDS) plots of mobile macrofauna community structures (MMCS) associated with *Sargassum* at Hydrostation S and nearby Sta. 1 a) MDS depicting MMCS of samples collected from Hydrostation S in 1972 and 1973 illustrating seasonal cyclicity (Butler et al. 1983) b) All Hydrostation S historical samples for 1971-1974 (n = 41) and recent sample (n = 1) from nearby Sta. 1. Note extreme disparity of taxonomic community structure recorded in 2012 vs. tight clustering of 41 samples documented from 1971-1974
