# Supplementary material for: Pelagic Sargassum community change over a 40-year period: temporal and spatial variability
Source: Mar Biol. 2014 Sep 14;161(12):2735–51. doi: 10.1007/s00227-014-2539-y (PMC4231207; doi:10.1007/s00227-014-2539-y)
Supplement: Supplementary file 3 — Supplementary material 3 (PDF 203 kb) [file 227_2014_2539_MOESM3_ESM.pdf]

Supplementary Table 2. Feeding categories assigned to mobile macrofauna. Where feeding modes could not be found for individual species, feeding categories were assigned based on published information about relatives where possible.

Pelagic *Sargassum* community change over a 40-year period: temporal and spatial variability. *Marine Biology*
C. L. Huffard*, S. von Thun, A. D. Sherman, K. Sealey, K. L. Smith, Jr.
*Corresponding author: Monterey Bay Aquarium Research Institute, 7700 Sandholdt Rd, Moss Landing, CA 95039; chuffard@mbari.org, phone: +1-831-775-1839, fax: (831) 775-1620

Taxon	Feeding category	Reference (direct or by extension from studies of relatives)
Xenacoelomorpha: acoela		
Heterochaerus sargassi	unknown	
Nemertea: Nematoda		
Nemoatode- unidentified	detritivore	Kito 1982
Platyhelminthes: Polycladida		
Acerotisa notulata	eats sessile fauna	Galleni et al. 1980
Chatziplana grubei	eats sessile fauna	Galleni et al. 1980
Platyhelminthes- unidentified	eats sessile fauna	Galleni et al. 1980
Platyhelminthes: Seriata		
Polycladus sp.	eats sessile fauna	Galleni et al. 1980
Platyhelminthes: Polycladida		
Gnesioceros sargassicola	eats sessile fauna	Galleni et al. 1980
Platyhelminthes: unidentified	unknown	
Annelida: Phyllodocida		
Myrianida sp.	detritivore	Giangrande et al. 2000
Platynereis dumerilii	herbivore	Hay et al. 1988
Polychaeta- unidentified	unknown	
Annelida: Spionida		
Spoinid worm- unidentified	detritivore	Taghon et al. 1980
Mollusca: Caenogastropoda		
Janthina janthina	eats coelenterates	Bieri 1966
Litiopa melanostoma	unknown	Butler et al. 1983
Gastropod- unidentified	unknown	
Mollusca: Littorinimorpha		
Rissoa sp.	detritivore	Wigham 1976
Mollusca:Mesogastropoda		
Bittium sp.	herbivore	Sureda et al. 2009
Mollusca: Nudibranchia		
Corambe obscura	eats sessile fauna	Zolotarev 1996
Cuthona sp.	eats sessile fauna	Folino 1997
Doto pygmaea	eats sessile fauna	Lambert 1991
Fiona pinnata	eats sessile fauna	Bieri 1966
Scyllaea pelagica	eats sessile fauna	Thompson and Brown 1981
Spurilla neapolitana	eats sessile fauna	Conklin and Mariscal 1977
Nudibranch "B" *sensu* Butler et al. 1983	eats sessile fauna	Lambert 1991
Nudibranch "D" *sensu* Butler et al. 1984	eats sessile fauna	Lambert 1991
Nudibranch- unidentified	eats sessile fauna	Lambert 1991

Arthropoda: Amphipoda		
Ampithoe spp.	herbivore	Skutch, 1926; Sotka 2003
Biancolina spp.	herbivore	McGrouther 1983
Deutella incerta	detritivore	Guerra-García and Tierno de Figueroa 2009
Gammarus sp.	herbivore	Orav-Kotta et al. 2009
Hemiaegina minuta	detritivore	Guerra-García and Tierno de Figueroa 2009
Hyale sp.	detritivore	Sotka 2003
Sunamphitoe pelagica	herbivore	Butler et al. 1983
Amphipod- unidentified	detritivore	Butler et al. 1983
Arthropoda: Copepoda		
Dactylopusia tisboides	detritivore	Butler et al. 1983
Copepod- unidentified	detritivore	Butler et al. 1983
Arthropoda: Decapoda		
Leander tenuicornis	eats mobile fauna	Butler et al. 1983; Johnson and Atema 1986
Hippolyte coerulescens	herbivore	Zupo 2001
Latreutes fucorum	eats mobile fauna	Butler et al. 1983; Johnson and Atema 1986
Portunus sayi	eats mobile fauna	Butler et al. 1983
Planes minutus	eats mobile fauna	Butler et al. 1983; Frick et al. 2004
Toseuma carolinensis	eats sessile fauna	Main 1987
Shrimp- unidentified	unknown	
Arthropoda: Harpacticoida		
Scutellidium longicauda	herbivore	Gunnill 1982
Arthropoda: Isopoda		
Bopyrus sp.	parasite	Dreyer and Wagele 2001
Carpias minutus	detritivore	Butler et al. 1983
Cirolana sp.	detritivore	Butler et al. 1983
Grapsicepon sp.	parasite	Markham 1977
Idotea metallica	omnivore	Gutow et al. 2006
Probopyrinella latreuticola	parasite	Markham 1977
Isopod- unidentified	unknown	
"Parasitic isopod"- unidentified	parasite	Butler et al. 1983
Arthropoda: Ostracoda		
Ostracod- unidentified	unknown	
Arthropoda: Pantopoda		
Anoplodactylus petiolatus	eats sessile fauna	Varoli 1994
Endeis spinosa	detritivore	Soler-Membrives et al. 2013
Pycnogonida- unidentified	unknown	
Arthropoda: Tanaidacea		
Zeuxo (Zeuxo) normani	herbivore	Nakaoka 2002
Tenaid- unknown	unknown	
Chordata: Thaliacea		
Salpa sp.	eats phytoplankton	Andersen 1985
Chordata: Teleostei		
Balistes sp.	eats mobile fauna	Turner and Rooker 2006
Caranx sp.	eats mobile fauna	Casazza and Ross 2008

Diodon sp.	eats mobile fauna	Palmer 1979
Histrio histrio	eats mobile fauna	Adams 1960
Syngnathus typhle	eats mobile fauna	Casazza and Ross 2008
Stephanolepis hispidus	eats mobile fauna	Clements and Livingston 1983
Fish- unidentified	unknown	

References:

Adams JA (1960) A contribution to the biology and postlarval development of the Sargassum Fish, *Histrio Histrio* (Linnaeus), with a Discussion of the *Sargassum* Complex. Bulletin of Marine Science 10: 55-82

Andersen V (1985) Filtration and ingestion rates of *Salpa fusiformis* Cuvier (Tunicata: Thaliacea): Effects of size, individual weight and algal concentration. Journal of experimental marine biology and ecology 87: 13-29

Bieri R (1966) Feeding preferences and rates of the snail, *Ianthina prolongata*, the barnacle, *Lepas anserifera*, the nudibranchs, *Glaucus atlanticus* and *Fiona pinnata*, and the food web in the marine neuston. Publications of the Seto Marine Biological Laboratory 14: 161-170

Butler JN, Morris BF, Cadwallader J, Stoner AW (1983) Studies of *Sargassum* and the *Sargassum* community. Bermuda Biological Station for Research St Georges

Casazza TL, Ross SW (2008) Fishes associated with pelagic Sargassum and open water lacking *Sargassum* in the Gulf Stream off North Carolina. Fishery Bulletin 106

Clements WH, Livingston RJ (1983) Overlap and pollution-induced variability in the feeding habits of filefish (Pisces: Monacanthidae) from Apalachee Bay, Florida. Copeia 1983: 331-338 doi 10.2307/1444375

Conklin EJ, Mariscal RN (1977) Feeding behavior, ceras structure, and nematocyst storage in the aeolid nudibranch, *Spurilla neapolitana* (Mollusca). Bulletin of Marine Science 27: 658-667

Dreyer H, Wagele JW (2001) Parasites of crustaceans (Isopoda: Bopyridae) evolved from fish parasites: molecular and morphological evidence. Zoology-Jena- 103: 157-178

Folino NC (1997) The role of prey mobility in the population ecology of the nudibranch *Cuthona nana* (Gastropoda: Opisthobranchia). American Malacological Bulletin 14: 17-26

Frick MG, Williams KL, Bolten AB, Bjorndal KA, Martins HR (2004) Diet and fecundity of Columbus crabs, *Planes minutus*, associated with oceanic-stage loggerhead sea turtles, *Caretta caretta*, and inanimate flotsam. Journal of Crustacean Biology 24: 350-355

Galleni L, Tongiorgi P, Ferrero E, Salghetti U (1980) *Stylochus mediterraneus* (Turbellaria: Polycladida), predator on the mussel *Mytilus galloprovincialis*. Marine Biology 55: 317-326 doi 10.1007/BF00393784

Giangrande A, Licciano M, Pagliara P (2000) The diversity of diets in Syllidae (Annelida: Polychaeta). Cahiers de biologie marine 41: 55-65

Guerra-García J, Tierno de Figueroa J (2009) What do caprellids (Crustacea: Amphipoda) feed on? Marine Biology 156: 1881-1890 doi 10.1007/s00227-009-1220-3

Gunnill FC (1982) Macroalgae as habitat patch islands for *Scutellidium lamellipes* (Copepoda: Harpacticoida) and *Ampithoe tea* (Amphipoda: Gammaridae). Marine Biology 69: 103-116 doi 10.1007/BF00396966

Gutow L, Strahl J, Wiencke C, Franke H-D, Saborowski R (2006) Behavioural and metabolic adaptations of marine isopods to the rafting life style. Marine biology 149: 821-828

Hay ME, Renaud PE, Fenical W (1988) Large mobile versus small sedentary herbivores and their resistance to seaweed chemical defenses. Oecologia 75: 246-252

Johnson BR, Atema J (1986) Chemical stimulants for a component of feeding behavior in the common gulf-weed shrimp *Leander tenuicornis* (Say). The Biological Bulletin 170: 1-10

Kito K (1982) Phytal marine nematode assemblage on *Sargassum confusum A*gardh, with reference to the structure and seasonal fluctuations Journal of the Faculty of Science Hokkaido University Series VI Zoology 23: 143-161

Lambert WJ (1991) Coexistence of hydroid eating nudibranchs: do feeding biology and habitat use matter? The Biological Bulletin 181: 248-260

Main KL (1987) Predator Avoidance in Seagrass Meadows: Prey Behavior, Microhabitat Selection, and Cryptic Coloration. Ecology 68: 170-180 doi 10.2307/1938817

Markham JC (1977) Distribution and systematic review of the bopyrid isopod *Probopyrinella latreuticola* (Gissler, 1882). Crustaceana 33: 189-197 doi 10.2307/20103215

McGrouther MA (1983) Comparison of feeding mechanisms in two intertidal gammarideans, *Hyale rupicola* (Haswell) and *Paracalliope australis* (Haswell) (Crustacea : Amphipoda). Marine and Freshwater Research 34: 717-726

Nakaoka M (2002) Predation on seeds of seagrasses *Zostera marina* and *Zostera caulescens* by a tanaid crustacean *Zeuxo* sp. Aquatic Botany 72: 99-106

Orav-Kotta H, Kotta J, Herkül K, Kotta I, Paalme T (2009) Seasonal variability in the grazing potential of the invasive amphipod *Gammarus tigrinus* and the native amphipod *Gammarus salinus* (Amphipoda: Crustacea) in the northern Baltic Sea. Biological Invasions 11: 597-608

Palmer AR (1979) Fish predation and the evolution of gastropod shell sculpture: experimental and geographic evidence. Evolution: 697-713

Skutch AF (1926) On the habits and ecology of the tube-building amphipod *Amphithoe rubricata* Montagu. Ecology 7: 481-502 doi 10.2307/1931173

Soler-Membrives A, Arango CP, Cuadrado M, Munilla T (2013) Feeding biology of carnivore and detritivore Mediterranean pycnogonids. Journal of the Marine Biological Association of the United Kingdom 93: 635-643

Sotka EE (2003) Genetic control of feeding preference in the herbivorous amphipod *Ampithoe longimana*. Marine Ecology Progress Series 256: 305-310

Sureda A, Box A, Deudero S, Pons A (2009) Reciprocal effects of caulerpenyne and intense herbivorism on the antioxidant response of *Bittium reticulatum* and *Caulerpa taxifolia*. Ecotoxicology and Environmental Safety 72: 795-801 doi http://dx.doi.org/10.1016/j.ecoenv.2007.12.007

Taghon GL, Nowell ARM, Jumars PA (1980) Induction of suspension feeding in spionid polychaetes by high particulate fluxes. Science 210: 562-564

Thompson TE, Brown GH (1981) Biology and relationships of the nudibranch mollusc *Notobryon wardi* in South Africa, with a review of the Scyllaeidae. Journal of Zoology 194: 437-444 doi 10.1111/j.1469-7998.1981.tb04592.x

Turner JP, Rooker JR (2006) Fatty acid composition of flora and fauna associated with *Sargassum* mats in the Gulf of Mexico. Marine Biology 149: 1025-1036

Varoli FMF (1994) Feeding aspects of *Tanystylum isabellae* and *Anoplodactylus stictus* (Pantopoda). Revista Brasileira de Zoologia 11: 623-627

Wigham GD (1976) Feeding and digestion in the marine prosobranch *Rissoa parva* (Da Costa). Journal of Molluscan Studies 42: 74-94

Zolotarev V (1996) The Black Sea ecosystem changes related to the introduction of new mollusc species. Marine ecology 17: 227-236

Zupo V (2001) Influence of diet on sex differentiation of *Hippolyte inermis* Leach (Decapoda: Natantia) in the field. Hydrobiologia 449: 131-140 doi 10.1023/A:1017553422113
